# Promiscuous and lineage-specific roles of cell cycle regulators in haematopoiesis

**DOI:** 10.1186/1747-1028-2-6

**Published:** 2007-02-12

**Authors:** Stephen S Myatt, Eric W-F Lam

**Affiliations:** 1 Cancer Research-UK labs, Department of Oncology, MRC Cyclotron Building, Imperial College London, Hammersmith Hospital Campus, Du Cane Road, London W12 0NN, UK

## Abstract

Haematopoietic cell number is maintained by a delicate balance between cell proliferation, differentiation and death. Gene knockout studies in mice have revealed the complex roles of cyclins, CDKs, and CDK inhibitors in regulating cell proliferation and differentiation in the haematopoietic system. These studies point to families of cell cycle regulators which display both redundant and unique roles within a lineage and developmental-stage specific manner. Moreover, the promiscuity of these cell cycle regulators is critical for haematopoietic cell proliferation and differentiation. In this review, we discuss the current evidence from mouse models that the complexity and multifarious nature of the haematopoietic system is critical for its form and function.

## Background

In complex, multi-cellular organisms, the interplay between cell proliferation, differentiation, and death determines cell number, tissue architecture and function. This dynamic equilibrium is best appreciated in tissues with high volumes of cell turnover, in particular the haematopoietic system [1,2]. The human haematopoietic system turns over approximately 10^11 ^blood cells everyday, and the daily loss of blood cells must be balanced by an equally high rate of cell proliferation. Moreover, proliferation must be coupled with cell differentiation to continuously reconstitute and replenish all different cell lineages of the haematopoietic system. In addition to this routine turnover, the haematopoietic system exhibits remarkable flexibility at times of physiological stress. For example, red blood cell numbers increase under conditions of hypoxia, while granulocyte, macrophage and lymphocyte populations expand during infection. In contrast, the uncontrolled proliferation of haematopoietic stem cells (HSC) and progenitors is symptomatic of hyperplastic diseases and leukaemia. Thus, despite the ability of haematopoietic cells to undergo rapid proliferation and differentiation entry into the cell cycle is under stringent control during haematopoietic cell development; preventing progenitor populations becoming exhausted as a result of continuous division by restricting entry into the cell cycle. Indeed, the optimal reconstitution of haematopoiesis is achieved by quiescent cells [3], whereas cells which have already entered the latter stages of the cell cycle are ineffective at engraftment [4]. Evidence from murine and primate studies also suggests that a pool of slowly cycling HSC may also exist [5-7]. Ultimately, the decision to proliferate or remain in a quiescent state is central to both the maintenance and immunological function of the haematopoietic system, and is a decision largely executed by cell cycle regulation.

### The cell cycle: G1/S phase transition

Cell proliferation requires entry into and successful progression through the cell division cycle [8,9]. The mammalian cell cycle can be divided into four phases wherein S phase (DNA-synthesis) and M phase (mitosis) are separated by two intervals, G1 (gap 1-between M and S) and G2 (gap 2-between S and M). Nevertheless, most cells are in a quiescent or resting phase termed G0. The transition through G1 into S phase of the cell cycle represents a critical period within which cells become committed to cell cycle progression versus growth arrest, becoming independent of growth factor stimuli, and as such is of particular importance to the haematopoietic system.

G1/S-phase stage transition is regulated by two families of G1 cyclins, the D-type cyclins (cyclin D1, D2, and D3) and the E-type cyclins (cyclin E1 and E2) [8] and their dependent kinases (CDK4, -6 and -2). CDK4 and CDK6, bind to and are activated by the D-type cyclins, while CDK2 activity is induced by the E-type cyclins [9]. The activities of the CDKs are regulated by two families of cyclin dependent kinase inhibitors (CKIs) the Cip/Kip family and the INK4 family. The Cip/Kip family, comprised of p21^Cip1^, p27^Kip1 ^and p57^Kip2 ^modulate the activities of cyclin D-, E- and A-dependent kinases (reviewed in [8]). The INK4 family, comprised of p15^INK4b^, p16^INK4a^, p18^INK4c^, and p19^INK4d ^specifically inhibit D-type cyclin associated CDK4 and CDK6 activity by formation of binary complexes which block active cyclin-CDK complex formation. The anti-proliferative activity of INK4 proteins is also in part due to competition with Cip/Kip proteins for binding to the D-type cyclin/CDK complex, releasing the Cip/Kip proteins to inhibit cyclin E-CDK2. Upon activation cyclin D-CDK4/6 and cyclin E-CDK2 kinases phosphorylate members of the retinoblastoma (Rb) protein family, pRb, p107, and p130 early and late in G1 respectively leading to the dissociation of Rb and E2F. The release of pRb-related proteins from E2F repression induces progression from G1 to S phase.

**Figure 1:**
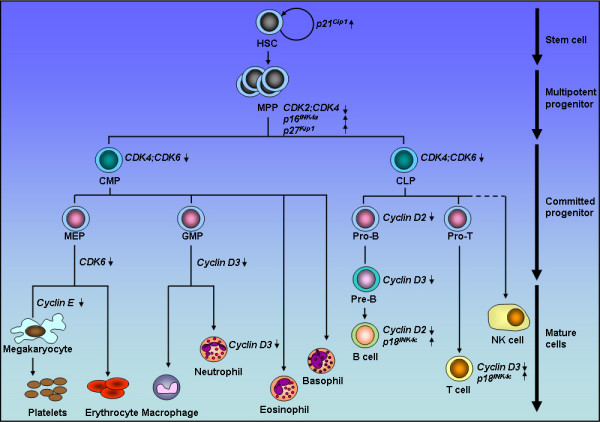
**Gene deletions affecting haematopoiesis**. Gene deletions are shown next to the cell type/pathway affected. The arrow indicates whether gene deletion results in an increase in cell number/activity/maturation or a decrease in cell number/activity/maturation. HSC, haematopoeitic stem cells; CMP, common myleoid progenitor; CLP, common lymphoid progenitor; MEP, megarkaryocyte/erythroid progenitor; GMP, granulocyte-macrocyte progenitor; CDK; cyclin-dependent kinase.

In single cell organisms, such as yeast, one cyclin dependent kinase (CDK) is enough to mediate cell cycle progression. However, concomitant with the increased levels of complexity and control required in multicellular organisms, mammalian cells have evolved families of cell cycle regulators, including multiple CDKs, cyclins, and CDK inhibitors (CKIs). The perceived rational for having multiple families of cell cycle regulators is that redundancy enables compensation where a family member becomes deregulated. However, differential expression patterns and activities of multiple family members may also allow for an increase in functional complexity, independent of their roles as guardians against pathway deregulation. Analysis of mice deficient of cell cycle regulators has provided evidence that cell cycle regulators exhibit both redundancy and specificity *in vivo *dependent on cell lineage and developmental timing. Moreover, studies on single as well as combined gene deletion mice have confirmed the overlapping functions of cell cycle regulators in haematopoietic cell proliferation and development, and demonstrated that the loss of function of a cell cycle regulator can be compensated by another family member (see Table 1). However, these studies have also revealed that some cell cycle regulators have unique roles, which cannot be substituted by other family members during haematopoiesis. Examples of single and double-deletion mutants generated in mice, and the cell type affected are shown in Figure 1. In the forthcoming review we summarize the evidence that D-type cyclins, CDKS and CKIs exhibit redundant, unique, and promiscuous roles in haematopoiesis.

**Table 1:** A summary of phenotypes observed following deletion of cell cycle regulating genes

**Gene deletion**	**Expression in haematopoieitc system**	**Haematopoietic system**	**Other phenotypes**
Cyclin D1	Unexpressed in the majority of haematopoietic lineages	No overt haematopoietic phenotype	Developmental neurological abnormalities; hypoplastic retina; impaired Schwann cell regeneration
Cyclin D2	Expression in majority of haematopoietic cell types; absent in small pre-B cells	Abnormal B-lymphocytes: impaired proliferation; hypo-responsive to BCR and mitogenic stimulation; impaired CD5 B cell development; immunodeficiency in IgG3 and IgA	Sterility in females, cerebellar abnormalities, hypoplastic testes in males; cerebellar abnoralities; reduced susceptability to specifc cancers
Cyclin D3	Expression in majority of haematopoietic cell types	Depletion of small pre-B cells; impaired thymic T cell development; impaired maturation of granulocytes in the bone marrow; reduced levels of circulating neutrophil granulocytes	Resistant to Notch-driven leukaemias
Cyclin D2; Cyclin D3	Upregulation of cyclin D3 in cyclin D2 null B cells; ubiquitous upregulaion of wild-type cyclin in single deletion embryos	As single deletions with severe megaloblastic anemia	Embryonic lethal at late developmental stages
CDK2	Expression in majority of haematopoietic cell types	No overt haematopoietic phenotype	Reduced body size; infertility
CDK4	Expression in majority of haematopoietic cell types	No overt haematopoietic phenotype	Dwarfism-like phenotype; infertility; hypocellularity in many organs; diabetes
CDK6	Expression in majority of haematopoietic cell types	Mild haematopoietic defects: hypoplasia of thymuses and spleens; delayed G1 progression in lymphocytes; depletion of megakaryocytes and erythrocytes	No overt phenotype
CDK2; CDK4	As above	Severe haematopoietic defects: reduced proliferation of multipotential progenitors; decrease in cellularity of all haematopoietic subpopulations	Embryonic lethality due to heart defects
CDK4; CDK6	As above	Multi-lineage haematopoietic abnormalities: reduction in cellularity of lymphoid, myeloid and granulocyte-macrophage progenitors; loss of mature haematopoietic cells	Late-stage embryonic lethal; anaemia
p15^INK4b^	Absent in HSC; increases in myeloid and lymphoid lineages	No overt haematopoietic phenotype	No overt phenotype
p16^INK4a^	Highly expressed in HSC; down-regulated with differentiation of all lineages	No overt haematopoietic phenotype; increased ability for clonal expansion of haematopoetic progenitor cells; long latency B-cell lymphomas	No overt phenotype
p18^INK4c^	Higher levels in HSC compared with more mature myeloid and lymphoid cells	Hyperplastic spleen and thymus; increased cellularity and hypersensitivity of T and B-cell lymphocytes to mitogenic stimulation; T-cell lymphoma	Widespread hyperplasia and organomegaly
p19^INK4d^	Higher levels in HSC compared with more mature myeloid and lymphoid cells	No overt haematopoietic phenotype	No overt phenotype
p21^Cip1^	Variable	Increase in HSC cycling; reduced progenitor cell replication; decrease of circulating inflammatory monocytes in peripheral blood	No overt phenotype
p27^Kip1^	Variable	Increased progenitor cell activity; hyperplasia observed in most haematopoietic organs, particularly pronounced in the thymus and spleen	Multiple organ hyperplasia
p57^Kip2^	Induced by TGF-β in specific progenitor/HSC (CB-CD34)	No overt haematopoietic phenotype	No overt phenotype

HSC; haematopoeitic stem cells, CDK; cyclin-dependent kinase.

### D-type cyclins

The D-type cyclins are the first components of the cell cycle machinery to become induced in response to mitogen stimulation, and provide a link between the extracellular mitogenic environment and the cell cycle. Two of the three D-type cyclins are usually expressed in every mammalian cell type, and the presence of multiple D-type cyclins is suggestive of functional redundancy. Indeed, the ablation of all D-type cyclins does not result in apparent defects in most other cell types and tissues until late in embryonic development (E17.5) where anaemia and cardiac defects are observed (see Table 1). Paradoxically, mouse deletion experiments have established that whilst loss of one or two of the D-type cyclins has little effect on the development of the haematopoietic system, simultaneous deletion of all D-type cyclins completely suppresses haematopoiesis, and prevents the expansion of haematopoietic stem cells [10]. These studies suggest that the inability of other cyclin-CDK kinase complexes to compensate for the loss of cyclin D activity is unique to the haematopoietic system. However, although these studies have established the overall importance of the D-type cyclin family in haematopoiesis, the specific roles of individual D-type cyclins are only beginning to emerge.

#### Cyclin D1

Cyclin D1 is not normally expressed in the majority of haematopoietic lineages, despite its collaborative role in haematopoietic stem cell expansion [10]. However, cyclin D1 expression has been detected in B-cell malignancies. In contrast, cyclin D2 and D3 co-express in a large number of haematopoietic cell types and are implicated in the proliferation and differentiation of most haematopoietic cells. Consistent with this hypothesis both cyclin D2 and D3 are essential for haematopoietic cell proliferation. However, cyclin D2 or D3 null mice do not demonstrate overt haematopoietic defects probably due to the fact that compensation exists between these two D-type cyclins.

#### Cyclin D2

Detailed analysis of different haematopoietic compartments has revealed unique tissue-specific roles for cyclin D2 in antigen-dependent B cell clonal expansion and CD5 B cell development. For example, B cells from *cyclin D2*^-/- ^mice demonstrate a specific requirement for cyclin D2 in B cell receptor(BCR)-induced proliferation [11,12]. Moreover, although conventional(B2) B cell development proceeds normally in cyclin D2 null mice, mature B cells in the spleens are hypo-responsive to BCR and mitogenic stimulation, and enter the cell cycle at a slower rate when compared with their normal counterparts in response to BCR stimulation [13]. Cyclin D2 is also important in CD5^+^(B1) B cell development; the CD5^+ ^B cell compartment in the peritoneal cavity is dramatically reduced in cyclin D2 deficient mice [13]. Cyclin D2 deficient mice also exhibited fewer B cell progenitors (Sca1^+^B220^+^) but normal levels of other haematopoietic progenitor cells [14]. In addition, clonal assays demonstrated that colonies from cyclin D2 null mice were less mature (CD19^lo^) than those from wild-type mice (CD19^hi^) indicating that cyclin D2 is rate-limiting for the production of B lymphoid progenitor cells whose proliferation does not depend on BCR signaling. Normal CD5^+^B cells are important for eliminating bacterial pathogens through secretion of IgG3 subtype antibodies, and functioning as precursors of IgA-secreting plasma cells. Thus, cyclin D2 null mice may be expected to be deficient in host defense against bacterial pathogens; indeed, *cyclin D2*^-/- ^mice display specific immunodeficiency in IgG3 as well as in IgA levels.

#### Cyclin D3

Cyclin D3 also has a unique role in B cell development. Analysis of cyclin D3 null mice showed that cyclin D3 was specifically required for the development of pre-B cells [15]. Consistent with this, cyclin D3, but not cyclin D2, is induced during the transition from pro-B cell to pre-B cell. Although in normal mice cyclin D2 is abundantly expressed in pro-B cells (CD43^+^B220^+^CD25^-^), large pre-B cells (CD43^lo^B220^+^CD25^lo^), and immature cells (B220^+^IgM^+^) from the bone marrow, it is absent in small pre-B cells (CD43^-^B220^+^CD25^+^). Indeed, cyclin D3 is the only D-type cyclin expressed in these small pre-B cells, and consequently small pre-B cells are depleted in cyclin D3 deficient mice.

Cyclin D3 also has a specific role in T cell development. Cyclin D3 deficient mice display a defect in thymic T cell development characterized by a significant decrease in CD4^+^CD8^+ ^double-positive (DP) T cells. This phenotype results from a non-redundant role for cyclin D3 in the maturation of CD4^-^CD8^- ^double-negative (DN) T cells, which are the precursors of DP T cells. In normal mice, developing DN T cells successively pass through four stages (DN-1 to DN-4), whereas cyclin D3 deficient thymocytes fail to undergo the proliferative burst that normally occurs during the DN-3 to DN-4 transition. In addition, cyclin D3 null mice also display defects in the maturation of granulocytes in the bone marrow and have reduced levels of circulating neutrophil granulocytes [16]. As a result, cyclin D3 null mice are unable to mount a normal response to bacterial infection. Yet, the development of the myeloid progenitors proceeds relatively normally in mice lacking cyclin D3, indicating that the cyclin D3 function is only required at later stages of neutrophil development.

#### Compensation and redundancy of cyclin D2 and D3

Together these studies demonstrate the importance of cyclin D2 and cyclin D3 in haematopoiesis. However, some questions remain unanswered; namely, cyclin D2 is essential for B cell development, yet why do cyclin D2 null mice have a normal number of conventional (B1) B cells? In fact, induction of cyclin D3 in cyclin D2 null mice has been found to compensate for loss of cyclin D2, and allow for the development of a normal number of mature B-lymphocytes [11]. In normal mature B cells cyclin D2 is the predominant D-type cyclin and cyclin D3 is not expressed at significant levels. However, in cyclin D2 null B cells, cyclin D3 is up-regulated to compensate for the loss of cyclin D2, and has been shown to complex with CDK6 in mitogen-stimulated cells. This was the first demonstration that loss of a D-type cyclin causes specific expression and functional compensation by another member of the family *in vivo*, and thus provides a rationale for the presence of mature B-lymphocytes in *cyclin D2*^-/- ^mice [11,14].

The compensation between different D-cyclins was further confirmed by double D-type cyclin deletion experiments, generating mice expressing only one cyclin D. These studies revealed that in single-cyclin D embryos, the tissue-specific expression patterns of D-cyclins were lost and the remaining D-cyclin was ubiquitously expressed [10]. These double D-type cyclin deleted mice did not show additional haematopoietic phenotypes compared with the single D-type cyclin deficient mice, except that cyclin D2 and -D3 deletion is embryonic lethal at late developmental stages (~E18.5), and cyclin D2 and -D3 null embryos developed severe megaloblastic anaemia [10].

### Cyclin-dependent kinases (CDKs)

CDK4 and -6 are the preferred catalytic subunits of cyclin D. Mice lacking the CDK4 gene display a dwarfism-like phenotype, infertility, and hypocellularity in many organs (see Table 1). However, no overt haematopoietic phenotype is observed in the CDK4-deficient mice, probably reflecting the compensation for loss of CDK4 by CDK6 [17]. In contrast, CDK6 null mice exhibit mild haematopoietic defects, including hypoplasia of thymuses and spleens, and depletion of megakaryocytes and erythrocytes [18]. Thus, it appears that CDK6 has a developmental and proliferative role specific for haematopoietic cells. Consistent with this notion, deletion of CDK6 results in delayed G1 progression in lymphocytes, but not in other cell types, such as mouse embryo fibroblasts.

Interestingly, mice defective for both CDK4 and CDK6 have a similar but milder phenotype compared with D-type cyclin deficient mice. CDK4 and -6 double mutant mice also display multi-lineage haematopoietic abnormalities, including a reduction in cellularity of lymphoid, myeloid and granulocyte-macrophage progenitors as well as a significant decrease in number of the more mature haematopoietic cells, such as monocytes, macrophage, lymphocytes and erythrocytes. Interestingly, further studies have revealed that D-type cyclins complex with CDK2 in the absence of CDK4 and -6, which may account for the less severe haematopoietic phenotype observed in cyclin-D null mice versus CDK4 and -6 null mice.

CDK2 is activated by E-type and A-type cyclins, and its activity is required for the transition through the G1/S and S phases. Unexpectedly, analysis of CDK2 deficient mice showed that CDK2 is dispensable for mouse development, suggesting functional compensation between different CDKs [19]. Although the *CDK2*^-/- ^mice have reduced body sizes, CDK2 null mice do not display any haematopoietic specific defects. However, consistent with the compensation for CDK4 by CDK2, double deletions of CDK4 and CDK2 results in the reduced proliferation of multipotential progenitors, culminating in a decrease in cellularity in all haematopoietic subpopulations [20]. These findings indicate that CDK4, not only has overlapping roles with CDK6, but that in the absence of CDK4, cyclin D may cooperate with CDK2 to control haematopoietic progenitor proliferation. Moreover, it suggests that only regulators for early G1, but not later cell cycle phases have unique roles for haematopoietic development. Indeed, mice null for E-type cyclins demonstrate largely normal development but severely impaired endoreplication of trophoblast giant cells and megakaryocytes; thought largely to reflect a failure to incorporate MCM helicase into DNA replication origins during G1/S phase progression [21,22]. However, whilst cyclin A1/2, cyclin B1/2/3 and cdc2(CDK1) are important cell cycle regulators for G2 and M phase progression, single gene deletion mice for these cell cycle regulators have no obvious haematopoietic phenotypes.

### The Cip/Kip family of CKIs

The Cip/Kip family of proteins are potent inhibitors of CDK2 kinases and may also stabilize cyclin D-CDK4/-6 complex formation. Despite p57^Kip2 ^being previously reported to be induced by transforming growth factor β (TGF-β) in primary human CD34^+ ^umbilical cord blood progenitor/stem cells (CB-CD34) [23], p21^Cip1 ^and p27^Kip1 ^are the predominant CKIs expressed in haematopoietic cells (see Table 1).

It is generally believed that p21^Cip1 ^and p27^Kip1 ^set the stoichiometric thresholds for cell cycle entry and progression through G1 in response to mitogenic stimuli, such as cytokines and interactions with stromal cells and the extracellular matrix. Indeed, recent evidence suggests that p21^Cip1 ^and p27^Kip1 ^are critical regulators of the increased HSC-specific entry into the cell cycle observed in FoxO deficient mice [24,25], implicating p21^Cip1 ^and p27^Kip1 ^in maintenance of HSC quiescence. Interestingly, phosphatase and tensin homologue (PTEN), which functions as a negative regulator of the phosphatidylinositol-3-OH kinase (PI(3)K)-Akt pathway, a known negative regulator of FOXO, may also restrict HSC activation [26].

Experimental evidence from p21^Cip1 ^and p27^Kip1 ^deficient mice suggests that p21^Cip1 ^and p27^Kip1 ^have specific roles in the regulation of quiescence of HSC and progenitors, respectively. For instance, p21^Cip1 ^knockout mice exhibit an increase in HSC cycling and exhaustion upon transplantation, supporting a role for p21^Cip1 ^in stem cell quiescence. Furthermore, p21^Cip1 ^may promote DNA repair, thereby maintaining stem cell integrity. In contrast, p27^Kip1 ^knockout mice display increased progenitor cell activity, and evidence suggests that whilst p27^Kip1 ^controls progenitor cell replication, it does not control HSC pool size [27,28]. Paradoxically, haematopoietic progenitor cells express p21^Cip1 ^at a high level, and although p21^Cip1 ^maybe sequestered from CDK2 in these cells, p21^Cip1 ^null mice display reduced progenitor cell replication, suggesting p21^Cip1 ^expression is required for progenitor cell replication. Interestingly, both the p21^Cip1 ^and p27^Kip1 ^deleted mice retain multi-lineage haematopoietic differentiation potential [29]. The mechanisms by which p21^Cip1 ^and p27^Kip1 ^gene inactivation differentially affects HSC and progenitors are not clearly understood. One possibility is that p21^Cip1 ^specifically binds and regulates cellular molecules through domains that are not found in p27^Kip1 ^and vice versa. Indeed, p21^Cip1 ^and p27^Kip1 ^differ substantially at their carboxy-terminus, and it is possible that domains located at this region confer specific functions [8].

Although the paradigm for Cip/Kip activity is to prevent cell cycle progression through inhibiting the CDK2 kinase activity, recent evidence demonstrated that CDK2 is dispensable for cell cycle inhibition mediated by the CKIs p27^Kip1 ^and p21^Cip1 ^as the loss of p27^Kip1 ^and p21^Cip1 ^confers similar proliferative advantages to cells with or without CDK2 [30]. These data suggests that either p27^Kip1 ^or p21^Cip1 ^have CDK2-independent cell cycle functions, or cells possess compensatory mechanisms that efficiently bypass their requirement for CDK2.

Aside from a decrease in HSC renewal capacity, mice lacking p21^Cip1 ^have no significant haematopoietic defects, except that they have a substantial decrease in inflammatory circulating monocytes in peripheral blood [31]. In contrast, mice lacking p27^Kip1 ^display multiple organ hyperplasia, with the most pronounced hyperplasia observed in the haematopoietic organs, such as thymus and spleen. Thymic hyperplasia in p27^Kip1 ^null mice was associated with increased T lymphocyte proliferation as a result of decrease in growth factor dependency. In the spleen, the absence of p27^Kip1 ^selectively enhanced proliferation of haematopoietic progenitor cells. *Ex vivo *proliferative assays also demonstrated that T and B lymphocytes lacking p27^Kip1 ^are hypersensitive to antigen receptor and/or mitogen stimulation. Thus, p27^Kip1 ^deficiency may cause an enhanced proliferation in response to mitogens in progenitors and more mature haematopoietic cells [32-34].

### The INK4 family of CKIs

The INK4 family have an essential role in G1 cell cycle phase progression through specifically inhibiting cyclin D-CDK4/6 kinase activity.

p16^INK4a ^is highly expressed in HSC, and down-regulated with differentiation of all lineages [35]. Loss of p16^INK4A ^*in vitro *increases the growth rate of myeloid colonies, and confers an increased ability for clonal expansion of hamatopoetic progenitor cells [36]. However, while it influences the growth and self-renewal kinetics of haematopoietic stem cells, p16^INK4a ^deficient mice do not exhibit abnormal haematopoiesis [37], suggesting its role in blood cell differentiation is not essential. Mice lacking p16^INK4a ^are not predisposed to cancer, except for a small percentage of animals that develop B-cell lymphomas after a long latency [38,39]. Furthermore, whilst p16^INK4a ^homozygous mutations are observed in 35% of acute lymphoblastic leukaemia (ALL) patients, p16^INK4a ^deletion is not an independent prognostic factor in ALL [40].

In contrast, p15^INK4b ^is absent in HSC, and its level increases in myeloid and lymphoid lineages [35,41]. Although hypermethylation of the p15^INK4b ^gene promoter region is a common event in acute myeloid leukaemia, transformation of myeloid cells by deregulated c-Myc is not thought to require inactivation of p15^INK4b ^[42]. However, as p15^INK4b ^is regulated by TGF-β1, which may maintain HSC quiescence independently of p21^Cip1 ^or p27^Kip1 ^[43], the role of p15^INK4b ^in stem cell fate requires further investigation. Interestingly, p15^INK4b ^null mice do not display gross developmental defects, except for extramedullary haematopoiesis and lymphoid hyperplasia in spleen and lymph nodes [44], suggesting that p15^INK4b ^has specific roles in regulating the proliferation of more mature haematopoietic cells.

p18^INK4c ^and p19^INK4d ^are expressed at higher levels in HSC compared with more mature myeloid and lymphoid cells [45]. In addition, the G1 arrest necessary for the terminal differentiation of B lymphocytes to immunoglobulin secreting plasma cells requires p18^INK4c ^[45-47]. In contrast, mice lacking p19^INK4d ^show normal haematopoiesis and both lymphoid and myeloid compartments differentiate normally [48]. Predictably, mice lacking p18^INK4c ^develop widespread hyperplasia and organomegaly similar to those developed by p27^Kip1 ^deficient mice demonstrating lymphoproliferative disorders and T cell lymphomas [44,49]. In addition, p18^INK4 ^deleted mice exhibit disproportionately enlarged and hyperplastic spleen and thymus. Although T and B lymphocytes develop normally in p18^INK4c ^deficient mice, they both exhibit increased cellularity and hypersensitivity to mitogenic stimulation.

Despite p15^INK4b ^and p18^INK4c ^single gene deletion mice having similar haematopoietic phenotypes, combined deletion of p15^INK4b ^and p18^INK4c ^does not result in a more severe phenotype, suggesting that p15^INK4b ^and p18^INK4c ^may function in different haematopoietic cell types and have cell-type specific functions [44]. Loss of p18^INK4c ^in T cells leads to hyperproliferation in response to T cell receptor (TCR) and/or co-receptor stimulation. These data suggest a model in which p18^INK4c ^functions as an inhibitory threshold in quiescent T cells and modulates proliferation of T cells [50]. Interestingly, p18^INK4c ^deleted HSC retain multilineage differentiation potential and are protected from exhaustion caused by p21^Cip1 ^deficiency, likely by a counteracting mechanism against cellular senescence, indicating that p18^INK4C ^has a role in restricting cell cycle entry and thus the pool size of HSC [51,52].

### Cooperation between Cip/Kip family of CKI and pRb-related proteins

Recent combined gene knock-out experiments have alluded to promiscuous roles of cell cycle regulators during haematopoiesis. The lack of overt proliferative phenotypes in the major haematopoietic organs of the *p27*^*Kip1*-/- ^mice indicates functional redundancy and compensation exists to prevent excess uncontrolled proliferation. Indeed, analysis of p27^Kip1 ^and p130 deficient mice have revealed that the pRb-related protein p130 can cooperate with p27^Kip1 ^to regulate the proliferation of a number of haematopoietic lineages [53].

In p27^Kip1 ^and p130 double deficient mice, the cellularity of the spleens but not the thymuses is significantly increased compared with their *p27*^*Kip1*-/- ^counterparts, affecting the lymphoid, erythroid and myeloid compartments [53]. In particular, *in vivo *cell proliferation is significantly augmented in the B and T cells, monocytes, macrophages and erythroid progenitors suggesting that p130 can compensate in part for the loss of p27^Kip1^. In fact, analysis of p27^Kip1 ^deficient splenocytes has shown that p130 can function as a CDK2 inhibitor and can compensate for the loss of p27^Kip1^. However, *p130 *and *p27*^*Kip1 *^double deficient thymocytes still lack a significant proliferative phenotype. This observation may be in part explained by the report that p107, like p130, can also interact with CDK2 in the spleen and thymus of p27^Kip1 ^null mice, suggesting that p107 may also be able to compensate for the absence of p27^Kip1 ^to modulate CDK2 activity. Interestingly, in the thymus the amount of p107 binding to CDK2 increases when p27^Kip1 ^is absent, suggesting that competitive binding of p107 and p27^Kip1 ^to CDK2 may exist.

Thus, this compensatory mechanism can help to explain the lack of a significant proliferative phenotype in the *p130 *and *p27*^*Kip1 *^double deficient thymocytes and possibly, for the absence of even more severe disorders in the splenocytes. Moreover, in the thymocytes, the expression level of p107 is high when compared with the splenocytes, thus allowing p107 to play a more prominent role than p130 in inhibiting CDK2 activity. Consequently, the lack of excessive thymocyte proliferation when both p130 and p27^Kip1 ^are lost may represent the high levels of p107 associated with CDK2 in the thymus but not the spleen [53]. It also appears that the relative levels of p107 or p130 expression in a specific tissue type dictates which of the pRb-related proteins cooperate with p27^Kip1 ^to regulate cell cycle entry and progression. This compensatory mechanism may provide an essential proliferation control in settings where p27^Kip1 ^is not expressed at normal levels, as is the case in many human tumours and hyperplastic conditions.

## Conclusion

**Figure 2:**
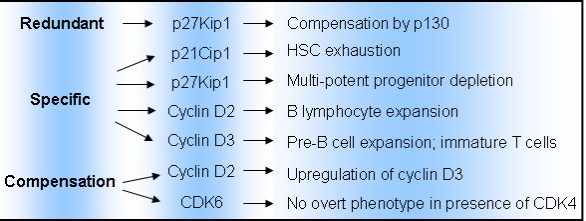
**Examples of cell cycle regulators exhibiting specificity, compensation, and redundancy during haematopoiesis**. Genes demonstrating specificity, compensation, and redundancy are shown with consequences for haematopoiesis.

It is now evident that the haematopoietic system displays a unique and specific dependency for cell cycle regulators, which is not unexpected, given the critical role of proliferation and differentiation in haematopoiesis. Furthermore, the cell cycle proteins involved in haematopoiesis almost exclusively regulate G1 entry and transition, probably attributable to the fact that the decision for cells to continue with cell cycle progression or to differentiate is often made in G1 phase [54]. A striking observation from mouse deletion experiments is that cell cycle regulators can exhibit specific, redundant, and compensatory activities, often, but not exclusively, linked to their temporal expression patterns during haematopoietic development (Figure 2).

Indeed, the lack of overt haematopoietic defects in the single and multiple cell cycle regulator gene deficient mice reveals that compensation and functional redundancy exist between members of individual cell cycle regulator family. In most circumstances, the masked phenotypes are results of the expression of more than one family member in a particular cell type. However, in some unique situations, the loss or deregulation of a cell cycle regulator leads to the compensatory induction of another family member, for instance, the compensation for loss of cyclin D2 by cyclin D3 in B cells described previously.

Evidence from the single and multiple gene deficient mouse studies also reveals specific non-overlapping roles for distinct cell cycle regulators such as p21^Cip1^, p27^Kip1^, and cyclin D2 during haematopoiesis. However, the majority of the present evidence has pointed to the tissue-specific expression patterns rather than specialised functions of cell cycle regulators as the basis for their cell type specific roles. For example, the specific requirement of cyclin D3 for pre-B cells is due to the fact that only cyclin D3 is expressed in these cells, and in addition, cyclin D2 can substitute for most functions of cyclin D1 in mice knock-in experiments [55]. However, in the case of p21^Cip1 ^and p27^Kip1^, their ability to regulate the self-renewal capacity and pool sizes of haematopoietic stem cells and progenitors, respectively, cannot be attributable to their cell type specific expression, as both are expressed in haematopoietic stem cells [1], and may reflect differential binding-specificities [8].

Most recent studies also reveal unexpected compensatory mechanisms, where the loss of one cell cycle regulator can be compensated by members of another family. For example, p130(RB2) has been shown to be able to substitute for the absence of p27^Kip1^. The fact that the haematopoietic phenotype in p130^RB2 ^and p27^Kip1 ^double mutant mice is stronger than that in p130^RB2 ^or p27^Kip1^-null mice indicates that their contribution to the phenotypes observed in p130^RB2 ^and p27^Kip1 ^double mutant mice is additive rather than a complete functional redundancy.

Ultimately, cell cycle regulators cooperate to set thresholds for cell cycle entry and exit in different lineages and cell types although there can be no doubt that functional redundancies between family members can protect cells from the consequence of deregulated cell cycle regulators. Redundancy may also serve to generate extra layers of control and complexity, which is required in multiorganisms. This allows specific developmental and proliferative clues to be channelled to distinct tissues and cells. In fact, the unique expression patterns and specific functions of these cell cycle regulators has provided specific functions for cell cycle regulators in haematopoiesis.

Intriguingly, most of the specific haematopoietic phenotypes only come to prominence after more detailed re-examination of the gene-deletion mice. As a consequence further evidence for specific and redundant roles of the cell cycle regulators in haematopoiesis is likely to be discovered. This information will provide crucial insights for devising new therapeutic strategies against the broad range of haematopoietic diseases.

## Competing interests

The author(s) declare that they have no competing interests.
